# Transient high-degree AV block in takotsubo syndrome

**DOI:** 10.1186/s43044-021-00182-5

**Published:** 2021-06-26

**Authors:** John E. Madias

**Affiliations:** 1 Icahn School of Medicine at Mount Sinai, New York, NY USA; 2 Division of Cardiology, Elmhurst Hospital Center, 79-01 Broadway, Elmhurst, NY 11373 USA

To the Editor:

I enjoyed very much reading the article by Revilla-Martí et al. [1], describing a 61-year-old man who suffered takotsubo syndrome (TTS) associated with transient high-grade atrioventricular (AV) block (HGAVB). The patient’s initial blood pressure of 90/60 is in keeping with early vagotonia seen in some patients in the setting of TTS [2, 3]; I wonder what was the heart rate (HR) of the patient’s sinus activity, which if slow would further support this attribution. The authors appropriately did not proceed with a pacemaker implantation, since the HGAVB was transient, with resolution prior to restoration of normal left ventricular function. I have some questions for the authors: (1) The QRS complexes in Fig. 1 appear wide (what was their width?), did they narrow in subsequent electrocardiograms (ECG) in the hospital and at follow-up? (2) Narrower subsequent QRS complexes would be supportive of the notion that the transient block was primarily at the His bundle level in addition to (or alternatively in the absence of) some impairment of the AV conduction, supported by the persisting long P-R interval at follow-up. (3) Were the QRS complexes narrower in a possibly available previous ECG, than the ones in the ECG of Fig. 1? (4) What were the HRs in such ECGs? (5) This will provide an explanation for the issue raised by the authors regarding the underlying pathophysiology (i.e., “supposed magnified sympathetic tone” and “ the apex” being “far away from the AV node, is the location of the most common wall segment motion abnormality” [1]), in the sense that the AV block was primarily or even exclusively related to transient intra-Hissian injury, engendered by the TTS-related tissue changes closer to areas of ventricular dyskinesis, rather than the remotely located AV node. (6) Was the P-R interval longer in the hospital than in subsequent ECGs at follow-up? (7) What were the HRs, P-R intervals, and QRS complex width in ECGs during hospital stay, discharge, and follow-up? (8) Are there any ECGs prior to the admission with TTS, showing a normal P-R interval and narrower QRS complexes than the ones seen in Fig. 1? (9) It is conceivable that diffuse spasm in small coronary branches could have caused ischemia in both the AV node and the Hiss bundle, or an increase in vagal tone could have affected the AV node. (10) TTS is associated with myocardial edema (ME), and one wonders about the effect of ME in the conduction velocity of the Hiss bundle, as a result of ME affecting it and/or the surrounding myocardium, wherein the Hiss bundle is embedded.

## Data Availability

Not applicable.

## Abbreviations

AV: Atrioventricular
ECG: Electrocardiogram(s)
HGAVB: High-grade AV block
ME: Myocardial edema
TTS: Takotsubo syndrome
